# Orienteering combines vigorous-intensity exercise with navigation to improve human cognition and increase brain-derived neurotrophic factor

**DOI:** 10.1371/journal.pone.0303785

**Published:** 2024-05-22

**Authors:** Emma E. Waddington, David J. Allison, Emilie M. Calabrese, Cara Pekos, Adrienne Lee, Jeremy J. Walsh, Jennifer J. Heisz

**Affiliations:** 1 Department of Kinesiology, Faculty of Science, McMaster University, Hamilton, Ontario, Canada; 2 Department of Physical Medicine and Rehabilitation, Schulich School of Medicine and Dentistry, Western University, London, Ontario, Canada; Bournemouth University, UNITED KINGDOM

## Abstract

Exercise enhances aspects of human cognition, but its intensity may matter. Recent animal research suggests that vigorous exercise, which releases greater amounts of lactate, activates more brain-derived neurotrophic factor (BDNF) in the hippocampus and, thus, may be optimal for supporting cognitive function. The cognitive benefits of exercise may be further augmented when combined with cognitive training. The sport of orienteering simultaneously combines exercise with spatial navigation and, therefore, may result in greater cognitive benefits than exercising only, especially at vigorous intensities. The present study aimed to examine the effects of an acute bout of orienteering at different intensities on cognition and BDNF compared to exercising only. We hypothesized that vigorous-intensity orienteering would increase lactate and BDNF and improve cognition more than moderate-intensity orienteering or vigorous exercise alone. Sixty-three recreationally active, healthy young adults (*M*_age_ = 21.10±2.75 years) with no orienteering experience completed a 1.3 km intervention course by navigating and exercising at a vigorous (80–85% of heart rate reserve) or moderate (40–50% of heart rate reserve) intensity or exercising vigorously without navigation. Exercise intensity was monitored using peak lactate, heart rate and rating of perceived exertion. Serum BDNF was extracted immediately before and after the intervention. Memory was assessed using the Mnemonic Similarity Task (high-interference memory) and the Groton Maze Learning Test (spatial memory). Both exercising and orienteering at a vigorous intensity elicited greater peak lactate and increases in BDNF than moderate-intensity orienteering, and individuals with higher peak lactate also had greater increases in BDNF. High-interference memory improved after both vigorous-intensity interventions but did not improve after the moderate-intensity intervention. Spatial memory only increased after vigorous-intensity orienteering, suggesting that orienteering at a vigorous intensity may particularly benefit spatial cognition. Overall, the results demonstrate the benefits of vigorous exercise on human cognition and BDNF.

## Introduction

As the brain ages, atrophy often outpaces plasticity, resulting in neurodegeneration and cognitive decline. Some brain regions are more susceptible to age-related decline than others, and the hippocampus is one of them [1]. After the age of 55 years old, the hippocampus atrophies at a rate of about 0.5% percent per year but progresses at twice that rate after the age of 70 years old [2] and nearly eight times that rate for individuals with Alzheimer’s disease [3]. This selective and severe hippocampal degeneration can impair critical hippocampal functions such as learning, memory, and spatial cognition and may compromise independent living [4]. Age is the greatest risk factor for dementia, and as the world’s population ages, dementia rates are predicted to climb sharply to affect over 152 million people by 2050 [5]. With no known cure for dementia, preventative measures that can help to stave off age-related cognitive decline are essential.

Exercise is one way to boost plasticity; however, emerging evidence suggests that not all forms of exercise are as effective. Vigorous exercise tends to evoke greater increases in plasticity through its stimulation of brain-derived neurotrophic factor (BDNF), a neurotrophic factor that supports the growth, function and survival of brain cells [6, 7]. Vigorous exercise has been associated with memory improvements in both younger [8] and older adults [9]. New research from animal models suggests that muscle-to-brain signalling during vigorous exercise is mediated by l-lactate (herein referred to as lactate), a product of pyruvate metabolism under anaerobic conditions that accumulates with increasing exercise intensity [10] and increases exponentially beyond the lactate threshold of ~ 4mmol/L of lactate in untrained adults [11, 12]. Although lactate has historically and erroneously been considered an inert metabolic waste [13], recent evidence points to its importance as both a fuel source [14] and an activator of BDNF [15–18] with rapid effects. Mere minutes after the initiation of vigorous exercise, lactate-activated BDNF has the potential to facilitate long-term potentiation within existing neural synapses to enhance neuroplasticity [19]. In this way, lactate accumulation during an acute bout of vigorous exercise may explain why acute exercise can immediately enhance certain cognitive functions [20]. To date, most research on the lactate-cognition connection has been done in animal models; only a few studies demonstrated the association in humans [10, 21–23]. Therefore, a primary objective of the present study was to examine the role of lactate in muscle-to-brain signalling on BDNF and cognition in humans.

We also wanted to examine whether the effects of vigorous exercise could be enhanced when simultaneously combined with a cognitively challenging task. During the process of neurogenesis, exercise predominantly impacts the proliferation of newborn neurons in the dentate gyrus, whereas cognitive training predominantly impacts the maturation and survival of those newborn brain cells [24]. Consequently, when combined, there is the potential for additive effects. Indeed, simultaneous exercise-cognition interventions in older adults improves cognition more than sequential interventions or cognitive training alone [25]. For example, older adults who engaged in spatial navigation while treadmill walking experienced enhancements in their spatial cognition more than older adults who only walked on the treadmill. Moreover, after four months of training, walkers saw a decrease in hippocampal volume, whereas navigators maintained a consistent volume, suggesting that there are added neurogenic benefits of combining exercise with navigation [26]. While intriguing, the mechanisms underlying these augmentative effects in humans are unclear, especially concerning the role that lactate and BDNF may play in promoting cognition, and testing those associations was the primary aim of this study.

For our simultaneous exercise-cognition training, we used the sport of orienteering, which naturally and simultaneously integrates exercise with spatial navigation and, therefore, may be an optimal way to combine exercise and cognitive training to target hippocampal plasticity and function [27]. The sport of orienteering requires the athlete to navigate through a series of checkpoints across an unknown terrain as fast as possible using only a topographical map and a compass [28]. Through focused attention and quick deduction of key information, highly skilled orienteers use spatial information and mental representations of an environment to navigate efficiently through space [28, 29], which is a critical function of the hippocampus [30]. Atrophy of the hippocampus impairs spatial navigation [31], and in cases of advanced AD, severe hippocampal degeneration renders the hippocampus unable to create, store, or use mental maps for wayfinding [32], causing disorientation even in familiar environments, a condition known as topographical disorientation [33, 34]. In line with the “use it or lose it” hypothesis [35], modern-day dependencies on vehicles for transport and passive navigation guided by Global Positioning Systems (GPS) cause most humans to underutilize their wayfinding abilities, leading to spatial memory deficits [36] and a reduced sense of direction [37] which orienteering has the potential to rescue. Moreover, to navigate through their environment, orienteers engage in various sensorimotor processes, and therefore, concepts of embodied cognition may also be relevant [38].

Indeed, our prior research revealed that orienteering experts aged 18–87 reported superior navigational strategies and better spatial memory than non-orienteering controls [27]. This recent observation resembles earlier research on London taxi drivers who, compared to controls, had a higher degree of navigational competency [39]. The taxi drivers also had a larger posterior hippocampus, a brain region primarily involved in supporting better visuospatial cognition, whose larger size was associated with greater years of experience [39–41]. However, not all parts of their hippocampus were larger; the anterior hippocampus, historically understood for its role in mediating episodic memory, was smaller in taxi drivers compared to controls, suggesting a trade-off between spatial and episodic memory that may be dependent on the training experience. Notably, the same trade-off was not seen with orienteering in that expert orienteers reported better spatial memory but not worse episodic memory to controls [27]. The simultaneous integration of exercise with navigation may be preventing the trade-off [27]. To date, only a handful of studies have examined the effect of orienteering training on cognition [42–44]; most have examined spatial cognition, and none have manipulated its intensity or examined lactate and BDNF.

Therefore, the present study aimed to examine the effects of orienteering at different exercise intensities (vigorous versus moderate) compared to vigorous intermittent exercise only on lactate, BDNF and different aspects of hippocampal-dependent memory. We hypothesized that the vigorous-intensity interventions would increase lactate more than the moderate-intensity intervention, resulting in a greater increase in BDNF and memory. Given the potential for additive effects of exercise-cognition training, we hypothesized that orienteering at a vigorous exercise intensity would elicit larger gains in BDNF and memory compared to orienteering at a moderate intensity or vigorous exercise alone.

## Methods

### Participants

Sixty-three participants (n = 41 female) who were healthy young adults (M_age_ = 21.10, SD = 2.75, range = 18–30) were recruited to the study using self-referral based on the criteria of being aged 18–30 years old and recreationally active (i.e., achieving at least 150 minutes up to 4.5 hours of recreational moderate-to-vigorous physical activity per week, as confirmed using the Physical Activity and Sedentary Behaviour Questionnaire; [45]. Recruitment was ongoing between July 2022 to May 2023. Participants were only included if they had engaged in orienteering from zero to a maximum of five times, a criterion based on previous research where an “orienteer” was defined as someone with at least six sessions of orienteering training [46]. Participants were screened to ensure eligibility using the following self-reported criteria: 1) no diagnosis of a neurological disorder or major health condition, 2) English language fluency, and 3) no colour blindness. Written informed consent was obtained through an online questionnaire. Participants were randomized into one of three groups: 1) moderate-intensity orienteering (n = 22), 2) vigorous-intensity orienteering (n = 21) and 3) vigorous-intensity exercise (n = 20), as described below. Participants received an honorarium of thirty Canadian dollars for their time. This study was reviewed and approved by the Hamilton Integrated Research Ethics Board (#14560) before recruitment and data collection.

### Materials and procedure

#### Baseline questionnaires

Following randomization and before the in-lab session, participants completed an online questionnaire (LimeSurvey software) to collect demographic information (see S1 Appendix).

All participants then completed the Physical Activity and Sedentary Behaviour Questionnaire [45] to assess their average weekly amount of moderate-to-vigorous aerobic exercise. The total activity amount was determined by multiplying the average length of an exercise session by the average number of active days (minutes/week).

The Navigational Strategy Questionnaire (NSQ) was used to assess participants’ baseline navigational tendencies [47]. Using a 5-point Likert scale, participants rated 44 items corresponding to three different navigational strategies: allocentric spatial processing, egocentric spatial processing, and procedural processing. For each strategy, an average score was calculated.

Baseline autobiographical memory was assessed using the Survey of Autobiographical Memory (SAM; [48]. In the SAM, subjective memory is evaluated across 26 items which are answered using a 5-point Likert scale. Each item is weighted and summed to obtain an average for four memory domains including episodic, spatial, semantic, and future memory. In this study, we examined episodic and spatial memory specifically.

#### Lab-based baseline measurements

In the lab, before the intervention, the participant’s height (centimetres), weight (kilograms), and waist circumference (in centimetres and taken from the anterior-superior iliac spine upon exhalation) were measured by a trained researcher.

Resting heart rate (HR_Rest_) was determined using a wetted Polar HR-10 chest heart rate (HR) monitor synchronized to a Polar Pacer Pro watch (Polar Electro Canada, Lachine, Quebec). The lowest HR value recorded in the final two minutes of a 12-minute supine resting period was used.

Maximum heart rate (HR_Max_) was estimated using the equation HR_Max_ = 208 - (0.7 * age) [49]. HR_Rest_ and HR_Max_ were used to calculate their exercising heart rate zones for the intervention using the percent of heart rate reserve (HRR) and the equation (HRMax−HR_Rest_) * (intensity) + HR_Rest_. For the moderate-intensity orienteering group, the exercise intensity range was calculated as 40–50% of HRR, and 80–85% of HRR was used for the vigorous-intensity orienteering and vigorous-intensity exercise groups.

Estimates of VO_2_ peak were calculated using the *WorldFitnessLevel*.*org* website [50]. Participants were asked to respond to the website questions as accurately as possible and input their anthropometric and HR measurements.

#### Intervention measures of exercise intensity

During the intervention, HR, ratings of perceived exertion (RPE) and lactate were recorded at the middle and end of the intervention course and 10 minutes post-intervention. The highest of these values was analyzed. Heart rate was recorded using the Polar HR-10 monitor. RPE was captured using the Borg 1–20 Scale [51]. Lactate was measured from a sample of whole blood obtained from the fingertip using the Lactate Plus portable analyzer (Nova Biomedical, Waltham, MA).

#### Pre- and post-intervention measurements

Before the intervention, the cognitive testing was completed before obtaining a serum sample for BDNF. Following the intervention, the blood sample was collected within 10 minutes of finishing the intervention course and was followed by cognitive testing.

For BDNF, three-hour fasted samples of venous blood were obtained from a vein in the antecubital fossa. Samples were collected into BD Vacutainer SST tubes (BD, Franklin Lakes, NJ), chilled on ice, allowed to clot for a minimum of 45 minutes following sample collection and then centrifuged at 1000 x g for 15 minutes at 4°C. For all samples, 300μL of supernatant was collected to obtain serum, aliquoted into microtubes, and stored immediately at -20°C until analysis. The concentration of serum BDNF was quantified using a sandwich Biosensis Mature BDNF Rapid^TM^ ELISA Kit (Biosensis Pty Ltd, Thebarton, Australia). Samples were diluted 100x and were run in duplicate. Using a BioTek SynergyMx spectrophotometer, absorbance was measured at 450 nm and analyzed using Gen 5 1.11 Software (BioTek Instruments Inc., Winooski, VT). Select samples whose concentration fell above the standard curve of the preliminary analysis were re-analyzed using a 125x dilution and the same protocol.

Memory was tested in two ways. First, memory was tested using Kirwan and Stark’s Mnemonic Similarity Task [52–54], a modified object recognition task that places a large emphasis on high-interference memory and hippocampal function. The Mnemonic Similarity Task begins with a study phase in which participants are shown a series of images of 60 everyday objects displayed on the screen for two seconds and must classify whether the image is an ‘indoor’ or an ‘outdoor’ item. This is immediately followed by a test phase, in which participants are shown 20 ‘repeat’ images (correct response = “old”), 20 ‘lure’ images that are highly similar but not identical to a previous image (correct response = “similar”), and 20 completely new, ‘foil,’ images (correct response = “new”) and asked to classify them. The Mnemonic Similarity Task has two measures which provide a valuable distinction between hippocampal-dependent high-interference memory and recognition memory. The “lure discrimination index” is a measure of high-interference memory, calculated as [*p* (“Similar” | Lure image)–*p* (“Similar” | Foil image)] × 100, and reflects one’s ability to correctly classify ‘lure’ items as “similar”. High-interference memory relies on the ability to remember specific details during encoding [55], which is dependent on the function of the hippocampus and is associated with hippocampal neurogenesis [54]. The second measure of the Mnemonic Similarity Task is general “recognition memory”, defined as the ability to correctly label a ‘repeat’ image as “old,” [*p* (“Old” | Repeat image)–*p* (“Old” | Foil image)] × 100. Recognition memory does not require participants to distinguish between highly interfering memories. It is less impacted by exercise and, therefore, theorized to be less dependent on hippocampal neurogenesis [56, 57]. The Mnemonic Similarity Task was administered before and after the intervention with different stimulus sets, and the order of each set was counterbalanced.

The second test of memory assessed spatial learning and memory using a computerized version of the Groton Maze Learning Test, adapted from the Milner Maze [58]. The 2D maze consists of a 28-step pathway hidden beneath a 10x10 grid of grey tiles that is revealed by clicking on adjacent tiles within the matrix using a mouse. If a correct tile in the sequence is selected, the tile briefly turns green, a rewarding auditory tone is played, and the participant can select a new tile. If an incorrect tile is selected, the tile briefly turns red, an incorrect auditory signal is played, and the participant must click on the previously correct tile before choosing a new tile. In the learning phase, participants complete the same maze five times in a row as fast as possible. To test delayed memory, participants complete the same maze once more following a 10-minute break. A Maze Efficiency Index [59] was calculated using the equation Maze Efficiency Index = number of correct moves per second/Log10 (time of the trial). Mean Maze Efficiency Index was calculated for the learning phase by averaging across the five learning trials whereas the Maze Efficiency Index for the test phase consisted of performance on the single test trial. The Groton Maze Learning Test was administered before and after the intervention with randomized maze sequences using computer software.

#### The intervention

The intervention started with a practice phase during which all participants completed a 500-meter outdoor practice course on the McMaster campus that included six orienteering checkpoints to get warmed up and familiarized with the intervention procedures. Participants learned how to read their HR on the Polar Pacer Pro GPS watch and maintain their pace so that their HR remained in the target range. Participants in the orienteering groups (both moderate and vigorous intensity) were taught how to use the orienteering map legend, orient their map, use the map to plan a route and locate checkpoints, and re-locate themselves should they make an error. Then, to simulate the intervention, the orienteering groups located the first three checkpoints at a light walking pace of 30–40% of HRR with the help of a researcher. For the fourth checkpoint, participants were encouraged to locate the checkpoint independently. For the final two checkpoints, participants located the checkpoints on their own and at their target intensity (moderate-intensity: 40–50% of HRR) or running (vigorous-intensity: 80–85% of HRR) pace. In contrast, participants in the vigorous exercise only group did not actively navigate. Instead, they followed a researcher around the 500 m course, beginning at a walking pace of 30–40% of HRR for the first four checkpoints and at their target intensity of 80–85% of HRR for the final two checkpoints.

Immediately after the practice phase, all participants were led to the start location of the intervention course by a researcher, and the Polar Pacer Pro GPS watch was started to track the participant’s HR and route. All participants completed the intervention course, which was approximately 1.3 kilometers and consisted of 10 checkpoints around the McMaster University campus according to their intervention condition. Those in the orienteering groups navigated to the checkpoints using the map at either a moderate (40–50% of HRR) or vigorous intensity (80–85% of HRR) along any route they chose. For safety reasons, a researcher silently followed participants during the intervention. For participants who were severely lost or had ventured outside the bounds of the orienteering map, the researcher informed them of their current location to ensure their safety but did not provide any additional information that would alter their navigational decisions. In contrast, those in the exercise only group exercised at a vigorous intensity (80–85% of HRR) but did not engage in orienteering. Instead, a member of the research team led the participant along the most efficient route.

All participants were responsible for tracking their HR at each checkpoint and were instructed to adapt the pace or pause until their HR returned to the target zone for a maximum of one minute. At the midpoint and finish checkpoints, a second researcher recorded HR, RPE and blood lactate.

### Statistical analysis

All data were analyzed using SPSS (IBM SPSS Statistics for Macintosh, version 28.0; IBM Corp., Armonk, NY). For all study variables, descriptive statistics were computed. Normality was assessed using skewness, kurtosis, and visual inspection of histograms. Data were screened for outliers using visual inspection of boxplots. For BDNF, cases were removed if BDNF concentration was above the standard curve, in which seven cases were removed (moderate orienteering = 1, vigorous orienteering = 4, vigorous exercise = 2). For the Mnemonic Similarity Task, three cases were removed as the difference in the percent corrected and raw score for appropriate key use was >8% (moderate orienteering = 2, vigorous orienteering = 1). Cases were also removed due to programming errors with the cognitive test software (Mnemonic Similarity Task: moderate orienteering = 1, vigorous orienteering = 1; Groton Maze Learning Test: moderate orienteering = 1, vigorous orienteering = 1) and because of errors in GPS data recording (vigorous orienteering = 2). Only complete cases were analyzed for each variable. All tests were computed with an alpha criterion of .05 and a 95% confidence interval.

#### Potential covariates and manipulation checks

To test for potential covariates, a one-way analysis of variance (ANOVA) was used to assess group differences in all demographic variables, weekly physical activity, spatial navigation tendencies and autobiographical memory, as well as pre-intervention differences in BDNF and cognition. To ensure that our intervention was adequate in reaching the desired exercise intensity, one-way ANOVA tests were computed for peak HR, peak RPE and peak blood lactate between groups. For blood lactate, a Kruskal-Wallis Means Ranks Test was used to confirm that the proportions of those above or below the lactate threshold of 4mmol/L differed by group, thus indicating that our intervention was adequate in reaching the desired exercise intensity.

#### Primary outcome measures

All primary outcome variables (BDNF, high-interference memory (lure discrimination index), recognition memory and spatial learning and memory efficiency) were analyzed using separate 2 x 3 mixed model ANOVAs with a within-subjects factor of time (pre, post) and between-subjects factor of group (moderate orienteering, vigorous orienteering, vigorous exercise). A *priori* one-sample *t-*tests (one-tailed) were computed to evaluate the pre- to post-intervention increases in BDNF and memory for each group with Hedge’s correction. Post hoc analyses of any between-group comparisons were performed with Bonferroni correction. Spearman’s correlation was used to evaluate the relationship between peak lactate and percent change in BDNF.

To further explore the relationship between peak lactate, percent change in BDNF and cognitive function, we computed a composite cognitive score was calculated by averaging the z-scores for the post-minus-pre change score values for each of our cognitive measures (high interference memory, recognition memory, Groton Maze learning efficiency, and Groton Maze test efficiency). Then, we performed an exploratory analysis using Spearman’s correlation to evaluate the relationship between the composite cognition score with peak lactate and percent change in BDNF. Finally, we conducted a partial Spearman’s correlation to determine whether the association between composite cognition score and peak lactate was diminished after controlling for the percent change in BDNF.

#### Secondary outcome measures

An exploratory analysis was done to quantify differences in the navigational performance of the two orienteering groups. The distance travelled by each of the orienteering groups (moderate orienteering, vigorous orienteering) was compared to the vigorous exercise group which, by design, travelled the most efficient route. A 2 x 3 mixed model ANOVA with a within-subjects factor of course half (start to midpoint, midpoint to finish) and between-subjects factor of group (moderate orienteering, vigorous orienteering, vigorous exercise) was used to identify group differences in distance travelled as indicated by the Polar Pacer Pro GPS watch. Post hoc analyses used a Bonferroni correction. Spearman’s correlation was used to determine existing associations between the total distance travelled and subjective measures of spatial processing/navigation and memory and for baseline measures of cognitive function for the two orienteering groups.

## Results

### Participants

Table 1 reports descriptive statistics of key baseline variables across groups. Ninety-two percent (n = 58/63) of participants were students at McMaster University. Participants did not differ in pre-exercise measures of high-interference or recognition memory or in spatial learning and memory. However, pre-intervention BDNF levels were higher for the moderate orienteering group than the vigorous orienteering or vigorous exercise groups (*p <* .001) (Table 2). Univariate ANOVA tests confirmed no other baseline differences between groups (Table 1).

**Table 1:** Descriptive statistics between intervention groups.

	Moderate Orienteering	Vigorous Orienteering	Vigorous Exercise
*n*	22	21	20
**Age (years)**	20.48 ± 2.34	21.76 ± 3.36	21.05 ± 2.46
**Age Range (years)**	18–28	18–30	18–26
**Sex (F/M)**	14/8	13/8	14/6
**Height (cm)**	170.16 ± 7.52	166.79 ± 8.53	169.40 ± 9.30
**Weight (kg)**	67.38 ± 10.53	68.91 ± 12.64	63. 91 ± 14.89
**WC (cm)**	82.52 ± 7.15	83.80 ± 7.72	81.97 ± 10.17
**Aerobic Physical Activity (min/week)**	172.74 ± 91.12	172.62 ± 99.64	198.25 + 103.76
Predicted VO_2_Max (mL/kg/min)	49.23 ± 6.58	50.19 ±6.23	48.95 ± 5.51
**Education**			
** < Secondary**	0%	0%	5%
** Secondary**	82%	57%	75%
** Post-Secondary**	18%	24%	5%
** Post-Graduate**	0%	19%	15%
**McMaster Student (No/Yes)**	1/21	2/19	2/18
**McMaster Campus Familiarity (%)**			
** Not Familiar**	5%	5%	5%
** Somewhat Familiar**	0%	5%	15%
** Neutral**	41%	24%	20%
** Fairly Familiar**	32%	29%	25%
** Very Familiar**	23%	38%	35%
**Orienteering Engagement (%)**			
** None**	77%	90%	80%
** 1–2 times**	18%	5%	15%
** 3–4 times**	5%	5%	5%
**Video Games (hours/week)**			
** None**	55%	62%	55%
** <1 to <3**	27%	24%	30%
** 3 to <7**	14%	10%	15%
** 7 to <9**	5%	5%	0%
**NSQ**			
** Egocentric**	3.34 ± 0.72	3.09 ± 0.61	3.02 ± 0.84
** Allocentric**	3.12 ± 0.65	2.97 ± 0.61	2.85 ± 0.79
** Procedural**	3.65 ± 0.47	3.64 ± 0.55	3.58 ± 0.68
**SAM**			
** Episodic**	100.68 ± 15.51	100.54 ± 12.32	102.53 ± 14.07
** Spatial**	98.21 ± 12.91	96.10 ± 14.01	97.10 ± 12.72

NSQ, Navigational Strategy Questionnaire; SAM, Survey of Autobiographical memory; WC, waist circumference. Values reflect M ± SD.

**Table 2:** Mean pre- and post-intervention values for primary variables.

	Moderate Orienteering	Vigorous Orienteering	Vigorous Exercise
*n*	19	19	20
** Recognition Memory**			
Pre (%)	87.89 ± 10.46	87.47 ± 8.02	85.20 ± 12.67
Post (%)	84.84 ± 9.95	85.21 ± 11.57	84.15 ± 11.91
** High-Interference Memory**			
Pre (%)	49.89 ± 26.22	47.68 ± 17.08	45.10 ± 22.27
Post (%)	42.37 ± 30.01	49.95 ± 18.55	52.20 ± 19.82
** Groton Maze Learning Efficiency**			
Pre	26.12 ± 10.32	23.28 ± 6.85	24.60 ± 5.89
Post	29.45 ± 7.88	29.42 ± 6.45	29.92 ± 4.52
** Groton Maze Test Efficiency**			
Pre	35.69 ± 14.24	30.62 ± 9.69	34.19 ± 10.68
Post	38.32 ± 11.07	36.24 ± 9.61	38.42 ± 9.23
*n*	21	17	18
** BDNF**			
Pre BDNF (ng/mL)	40.96 ± 10.15	38.85 ± 9.30	29.00 ± 6.19
Post BDNF (ng/mL)	42.33 ± 9.75	39.78 ± 8.71	31.33 ± 5.39

BDNF, Brain-derived neurotrophic factor. Values reflect M ± SD.

### Intensity manipulation checks

**Fig 1:**
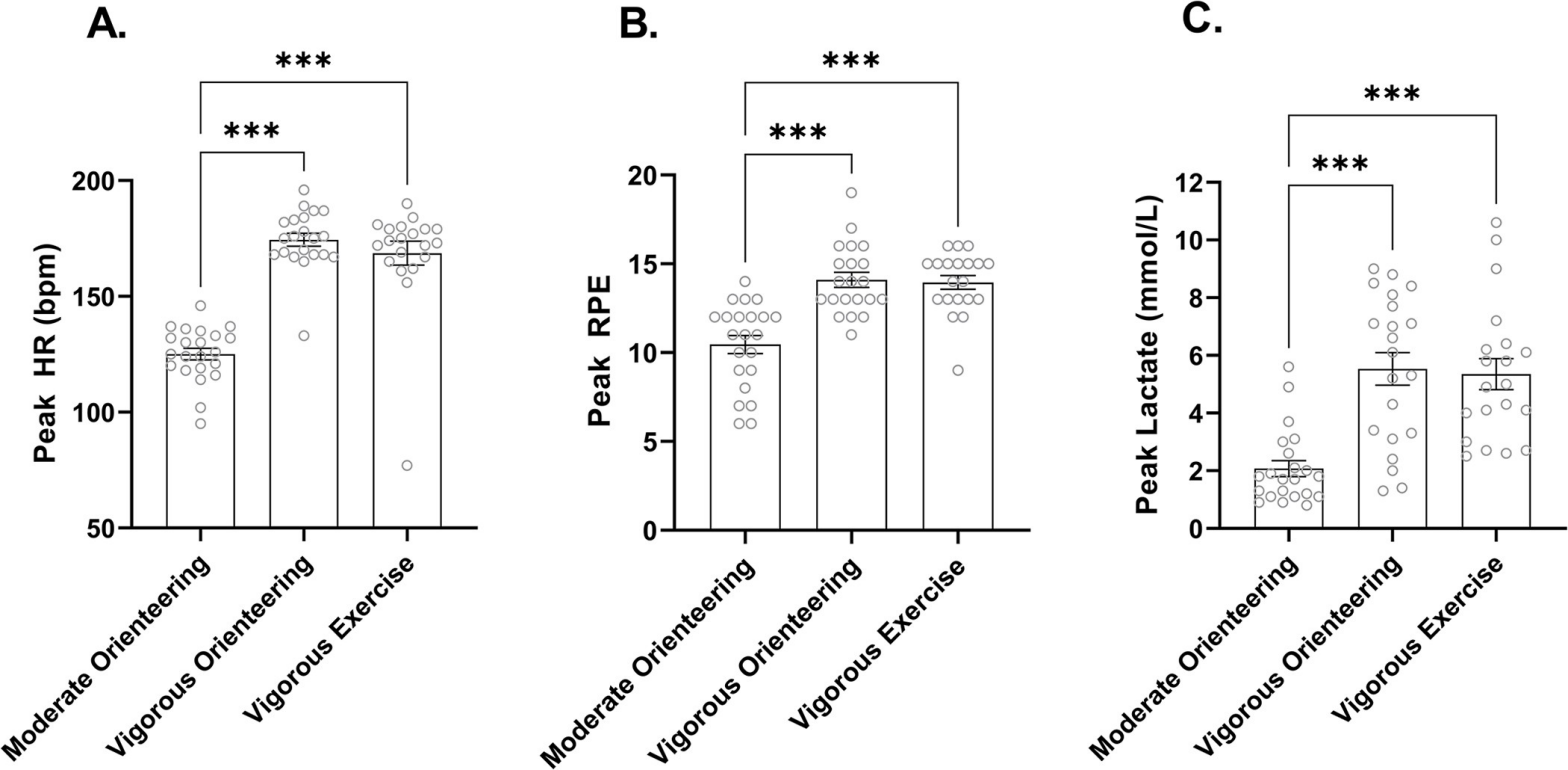
Group differences in exercise intensity metrics. (A) Peak HR, (B) peak RPE and (C) peak lactate achieved during the intervention between groups. Bars reflect mean score, and error bars represent ± SEM. *** = *p* < .001.

**Fig 2:**
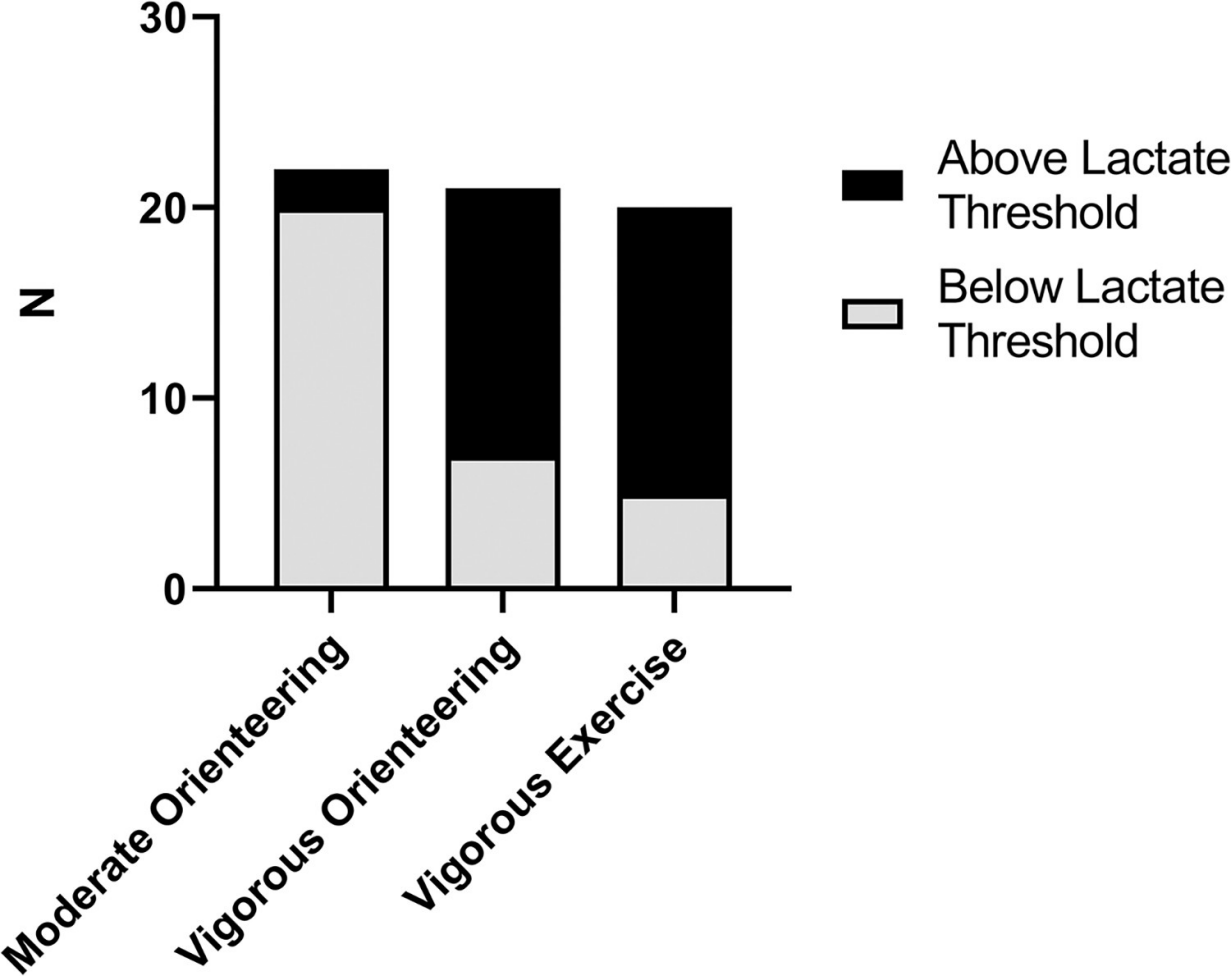
Proportion of intervention group above or below lactate threshold. Values reflect the number of participants per group with peak lactate above the lactate threshold of 4mmol/L.

Our intervention successfully induced the appropriate level of exercise intensity for each group, as confirmed by a significant main effect of group for peak lactate, *F*(2, 60) = 17.49, *p* < .001, *η*^*2*^ = .37, peak RPE *F*(2, 60) = 21.56, *p* < .001, *η*^*2*^ = .42, and peak HR *F*(2, 60) = 57.26, *p* < .001, *η*^*2*^ = .66. Post hoc comparisons indicate that the moderate orienteering group had lower peak HR, RPE and lactate than the vigorous orienteering and vigorous exercise groups which did not differ from each other (Fig 1). Peak HR was within the instructed range of 40–50% of HR_Max_ for the moderate-intensity group and 80–85% of HR_Max_ for the vigorous-intensity groups. The proportion of participants above the estimated LT of 4mmol/L differed significantly between groups, *H*(2) = 21.70, *p* < .001, with more participants above the LT in the vigorous orienteering and vigorous exercise groups than the moderate orienteering group (Fig 2).

### Primary outcomes

#### BDNF

**Fig 3:**
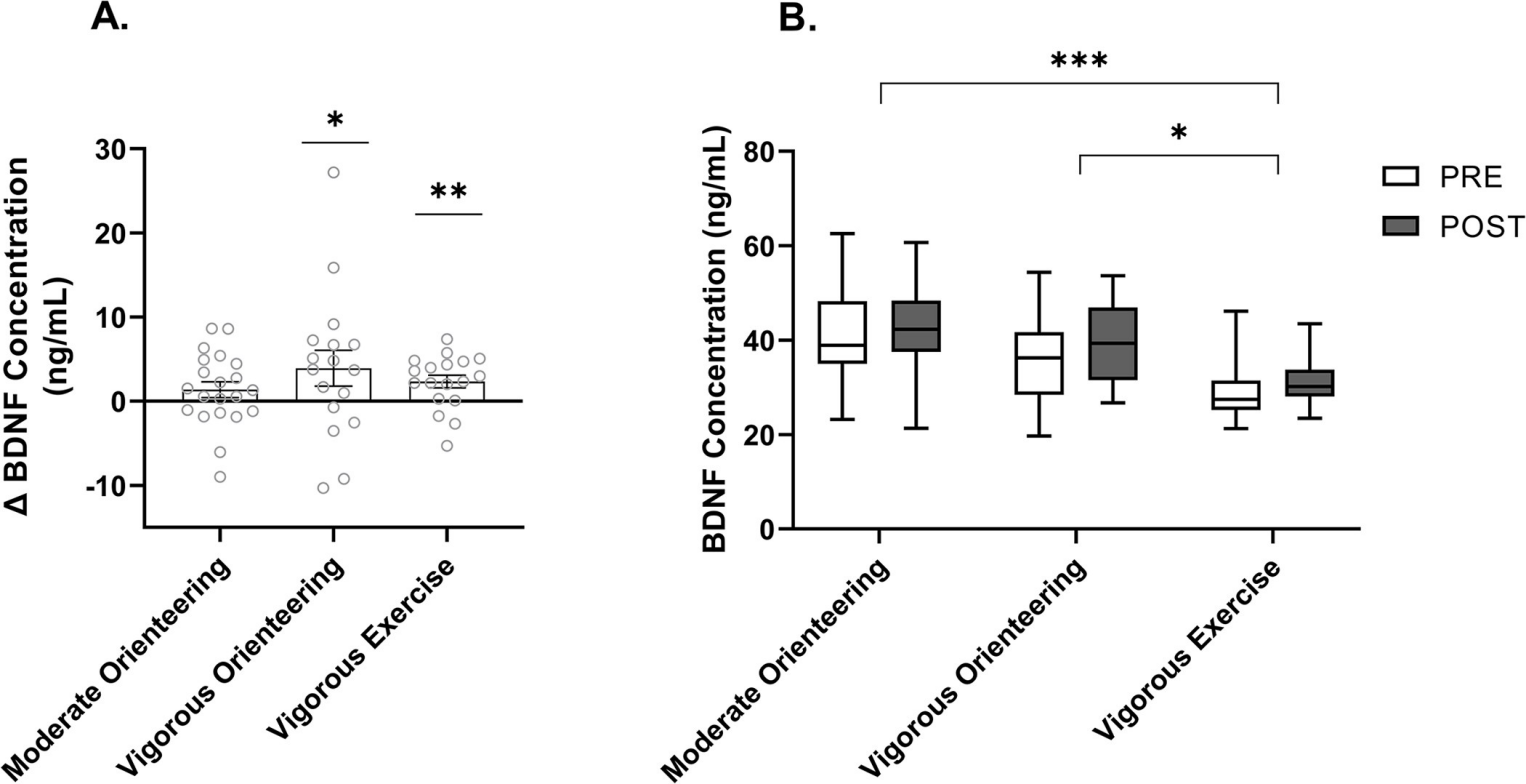
Change in BDNF concentration and group differences following intervention. (A) Bars reflect mean change in BDNF concentration between groups, error bars represent ± SEM. (B) A boxplot showing the interquartile range, median, minimum, and maximum concentration of BDNF between groups from pre- to post-intervention * = *p* < .05, ** = *p* < .01, *** = *p* < .001.

Fifty-six complete cases were analyzed (moderate orienteering: n = 21, vigorous orienteering: n = 17, vigorous exercise: n = 18). The mixed model ANOVA revealed a significant main effect of time, *F*(1, 53) = 10.51, *p* = .002, *η*_*p*_^*2*^ = .17, and group, *F*(2, 53) = 10.15, *p* < .001, *η*_*p*_^*2*^ = .28, but no interaction. Fig 3A shows an increase in BDNF for all groups over time, but the change was only significant for the vigorous orienteering, *t*(16) *=* 1.83, *p* = .043, *g* = .42, and the vigorous exercise groups, *t*(17) *=* 3.09, *p* = .003, *g* = .70, but not the moderate orienteering group, *t*(20) *=* 1.43, *p* = .084, *g* = .30. The vigorous exercise group’s BDNF levels were significantly lower at baseline and post-intervention (Table 2) than the other two groups (moderate orienteering, *p* < .001; vigorous orienteering, *p* = .020) (Fig 3B). This suggests that the group differences seen here reflect baseline differences that are unrelated to the intervention, and because of this, the relative change score (i.e., percent change) for BDNF was used in the correlation and mediation analyses below.

**Fig 4:**
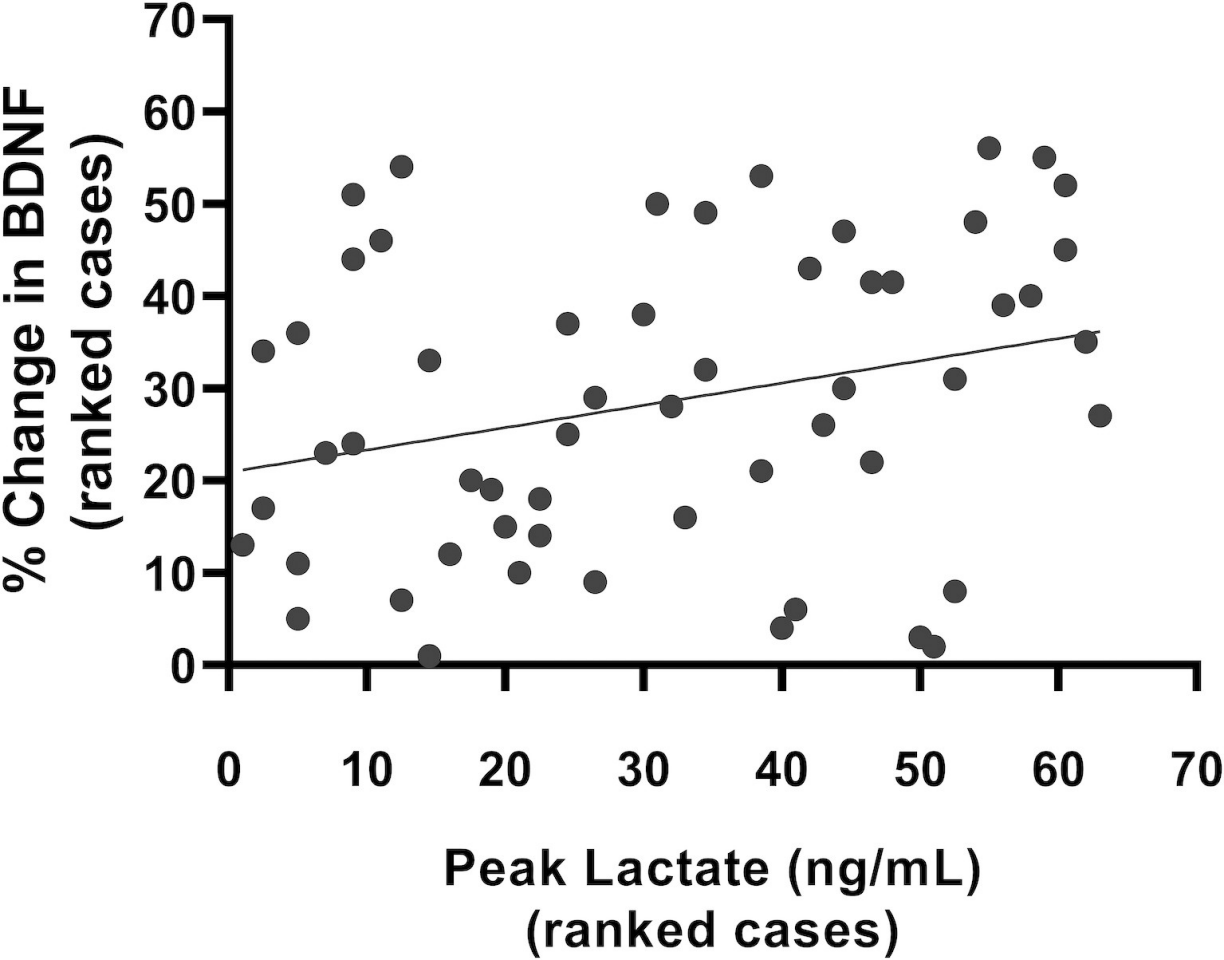
Correlation between peak lactate and percent change in BDNF. A scatterplot of ranked cases showing the correlation between the percent change in BDNF, and the peak lactate achieved during exercise. Y = 0.242x + 20.89, R = 0.28, *p* = .037.

Fig 4 depicts the results from the Spearman’s correlation, whereby a higher peak lactate achieved during exercise significantly correlated with a greater percentage increase in BDNF, *r*_*s*_ (54) = .28, *p* = .037.

#### High-interference and recognition memory

**Fig 5:**
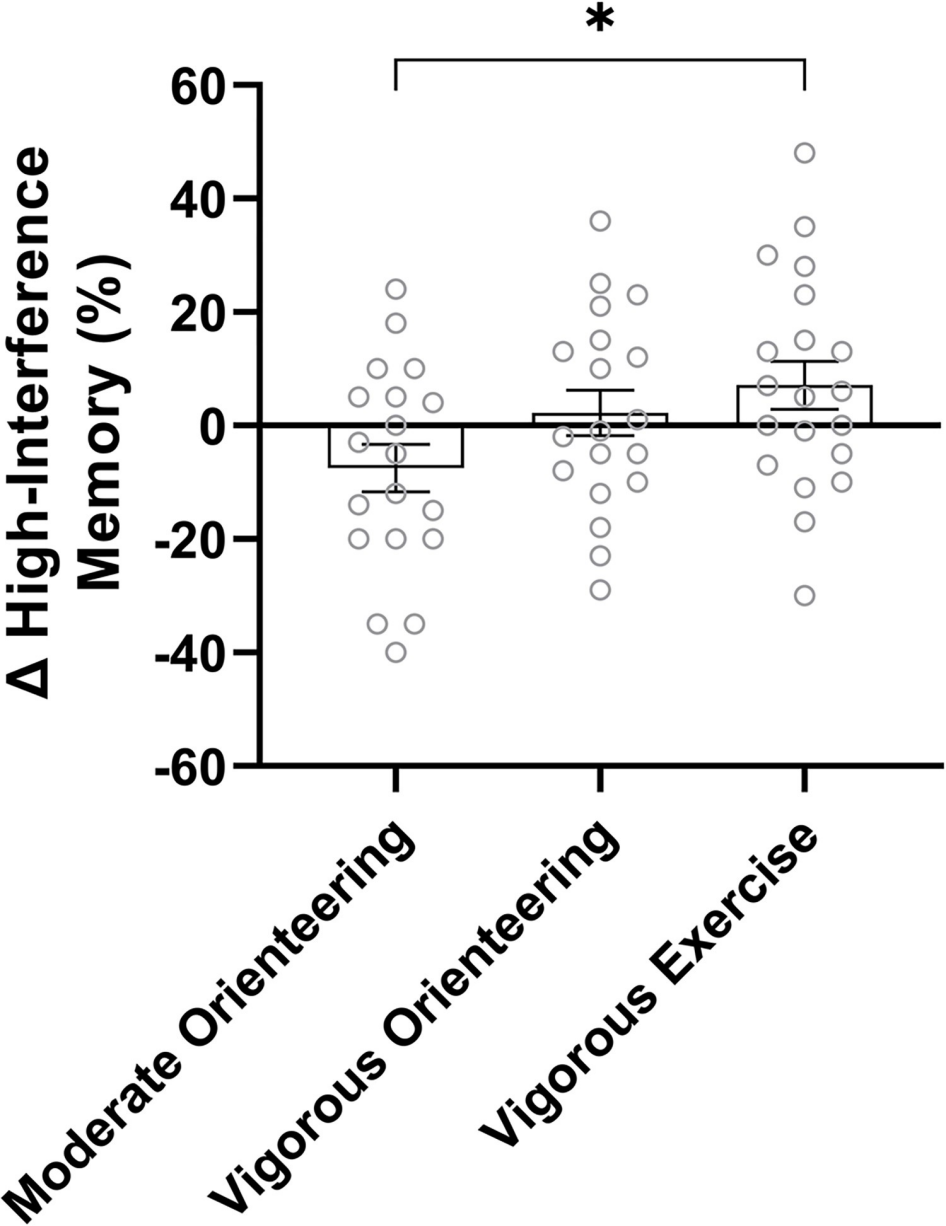
Change in high-interference memory following intervention. Bars reflect mean change in performance on the lure discrimination index measure of the Mnemonic Similarity Task between groups, and error bars represent ± SEM. * *= p* < .05.

Fifty-eight cases were included in the analysis (moderate orienteering: n = 19, vigorous orienteering: n = 19, vigorous exercise: n = 20). For high-interference memory, there was a significant group by time interaction, *F*(1, 55) = 3.23, *p* = .047, *η*_*p*_^*2*^ = .11. As shown in Fig 5, high-interference memory performance improved for the vigorous orienteering and vigorous exercise groups but declined for the moderate orienteering group. The difference between the moderate orienteering and vigorous exercise groups was significant, *t*(37) *=* -2.45, *p* = .019, *g* = -.77. There were no other effects for high-interference memory and no effects or interaction for recognition memory (Table 2).

#### Spatial learning and memory

**Fig 6:**
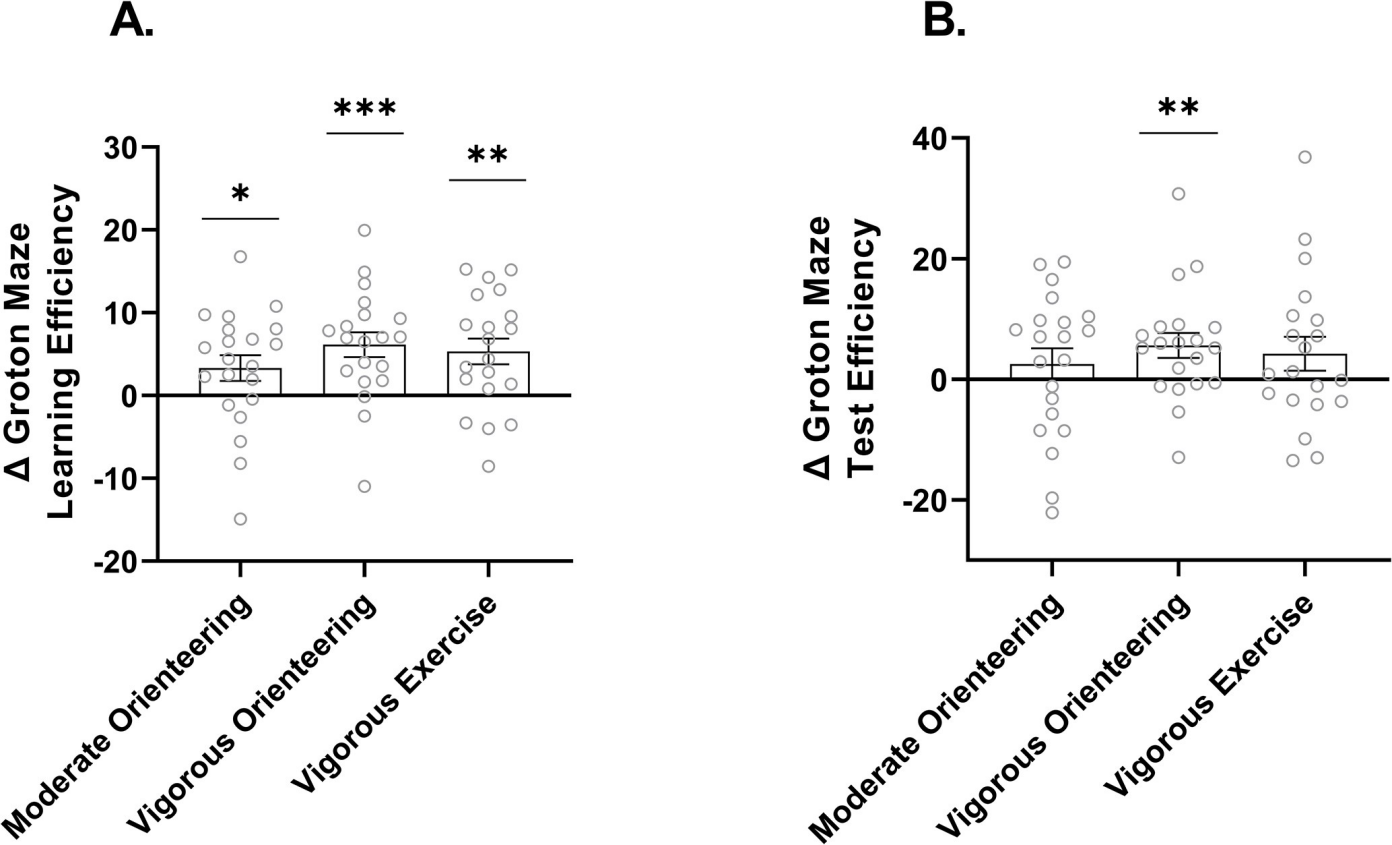
Change in spatial learning and memory following intervention. (A) Bars reflect mean change in Groton Maze learning efficiency by group. (B) Bars reflect mean change in Groton Maze test efficiency by group. Error bars represent ± SEM. * = p < .05, ** = p < .01, *** = p < .001.

Sixty-one complete cases were analyzed (moderate orienteering: n = 21, vigorous orienteering: n = 20, vigorous exercise: n = 20). Both the learning and delayed test trials of the Groton Maze Learning Test revealed a significant main effect of time for both the learning phase, *F*(1, 58) = 30.39, *p* < .001, *η*_*p*_^*2*^ = .35, and test phase, *F*(1, 58) = 8.09, *p* = .006, *η*_*p*_^*2*^ = .12, suggesting that all groups improved in spatial processing efficiency post-intervention (Table 2). For learning trials, Fig 6A depicts a significant improvement in performance for all groups following the intervention, though the largest effect size was for the vigorous orienteering group, *t*(19) = 4.11, *p* < .001, *g* = .88, followed by the vigorous exercise group, *t*(19) = 3.43, *p* = .001, *g* = .74, and the moderate orienteering group, *t*(20) = 2.14, *p* = .022, *g* = .45. For the delayed test performance (Fig 6B), only the vigorous orienteering group improved significantly, *t*(19) = 2.70, *p* = .007, *g* = .58. There was no effect of group or interaction for either the learning or delayed test trials.

#### Peak lactate, BDNF and cognitive function

In this exploratory analysis, the Spearman’s correlation revealed significant correlations such that greater improvements in composite cognition scores were associated with higher peak lactate, *r*_*s*_ (54) = .26, *p* = .049, and greater increases in BDNF, *r*_*s*_ (47) = .29, *p* = .041. However, after controlling for the percent change in BDNF, the association between cognition and peak levels of lactate obtained during exercise was no longer significant, *r*_*s*_ (44) = .21, *p* = .143.

### Secondary outcomes

**Fig 7:**
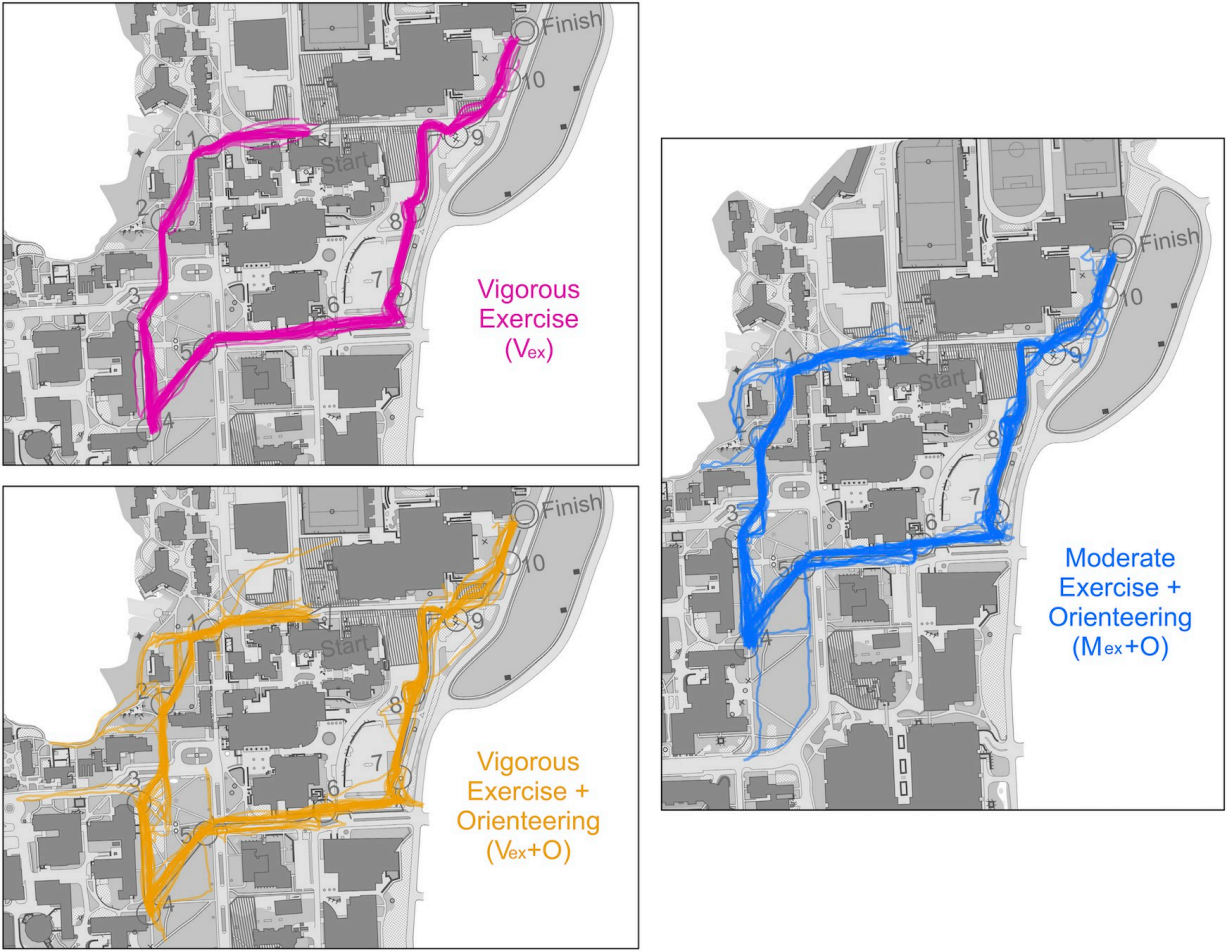
Routes traversed on the intervention course between groups. Figures show the routes traversed by each study group along the (approximately) 1.3 km intervention course around the McMaster University campus. Routes in pink show the paths of those in the vigorous exercise group (n = 20) who followed a researcher throughout the course at a running speed. These pink routes indicate the most efficient route. Routes in yellow show the paths of the vigorous orienteering group (n = 19) who actively navigated the intervention course at a running speed. The blue routes are those in the moderate orienteering group (n = 22) who navigated the intervention course at a walking speed. All routes were tracked using a Polar Pacer Pro GPS watch. Note that all participants started and finished in the same location, and checkpoints remained in the same location for all trials; any major differences in routes, such as a different starting location (seen in vigorous orienteering group map) can be attributed to GPS accuracy.

Sixty-one complete GPS cases were analyzed (moderate orienteering, n = 22, vigorous orienteering, n = 19, vigorous exercise, n = 20). The mixed model ANOVA for distance travelled revealed a significant main effect of group (*F*(2, 58) = 8.81, *p* < .001, *η*_*p*_^*2*^ = .23) such that the orienteering groups travelled longer (moderate orienteering: *p* = .018; vigorous orienteering: *p* < .001) than those in the non-orienteering group, but the orienteering groups did not differ (*p* = .509). Distances travelled can be found in Table 3. Fig 7 depicts the extra distance travelled by the two orienteering groups compared to the most efficient route.

**Table 3:** Distances travelled on the intervention course between groups.

	Moderate Orienteering	Vigorous Orienteering	Vigorous Exercise
*n*	22	19	20
**Average Distance Start to Midpoint (m)**	678.18 ± 136.40	718.42 ± 130.74	606.5 ± 13.09
**Average Distance Midpoint to Finish(m)**	681.82 ± 38.62	697.37 ± 57.24	641.00 ± 13.34
**Average Total Distance (m)**	1360 ± 149.83*	1415.79 ± 162.25***	1247.50 ± 17.73

Values reflect M ± SD.

* = *p* < .05 compared to the vigorous exercise group

** = *p* < .01 compared to the vigorous exercise group

*** = *p* < .001 compared to the vigorous exercise group.

Across participants in the orienteering groups, those who travelled farther and, by extension, made more errors reported significantly worse egocentric spatial processing tendencies from the NSQ *(r*_*s*_ (39) = -.44, *p* = .004) and subjective spatial memory from the SAM (*r*_*s*_ (39) = -.44, *p* = .004). They also performed significantly worse on the delayed test phase of the Groton Maze Test at baseline *(r*_*s*_ (37) = -.35 *p* = .030). Though not significant, allocentric spatial processing trended in the same direction (*r*_*s*_ (39) = -.27, *p* = .086). In contrast, procedural spatial processing (*r*_*s*_ (39) = -.13, *p* = .438) and subjective episodic memory (*r*_*s*_ (39) = .05, *p* = .744) were not related to the total distance travelled (Table 4), nor were any other aspects of cognition measured at baseline (Table 5).

**Table 4:** Correlation matrix between distance travelled in the intervention course by the orienteering groups and subjective measures.

	1	2	3	4	5	6
**1. Total Distance (m)**	-					
**2. NSQ Egocentric**	-.44 **	-				
**3. NSQ Allocentric**	-.27	.55 ***	-			
**4. NSQ Procedural**	-.13	-.32 *	-.02	-		
**5. SAM Episodic**	.05	.06	-.11	-.14	-	
**6. SAM Spatial**	-.44 **	.59 ***	.49 ***	-.26	-.11	-

NSQ, Navigational Strategy Questionnaire; SAM, Survey of Autobiographical Memory.

* = *p* < .05

** = *p* < .01

*** = *p* < .001.

**Table 5:** Correlation matrix between distance travelled in the intervention course by the orienteering groups and baseline measures of cognitive function.

	1	2	3	4	5
**1. Total Distance (m)**	-				
**2. High-Interference Memory Pre**	-.20				
**3. Recognition Memory Pre**	-.12	.14			
**4. Groton Maze Learning Efficiency Pre**	-.29	.20	.08		
5. Groton Maze Test Efficiency Pre	-.35*	.20	.04	.88***	

* = *p* < .05

*** = *p* < .001.

## Discussion

The present study was the first to examine the effects of an acute bout of orienteering versus exercise on cognition in a sample of healthy young adults who were recreationally active but unfamiliar with orienteering. The results revealed a strong effect of exercise intensity such that the vigorous-intensity interventions in the form of either running or orienteering elicited greater increases in lactate, BDNF and memory than the moderate-intensity intervention. Additionally, vigorous orienteering improved spatial learning and memory more than vigorous running, suggesting an additional benefit of simultaneous training.

This study demonstrates a link between lactate, BDNF and cognition in humans. A novel and important finding is that the higher peak lactate induced by our vigorous exercise interventions was associated with greater percent increases in BDNF and better memory than our moderate-intensity intervention, lending support for the hypothesis that lactate mediates muscle-to-brain signalling [10, 15, 16, 19]. Cognition was also significantly related to peak levels of lactate obtained during exercise. Interestingly, when controlling for BDNF, the relationship between cognition and lactate was no longer significant. We hypothesize that BDNF may partly underlie the effects of lactate on cognition, however, further work is needed to understand how exercise-induced lactate impacts cognition through and beyond its effects on BDNF [10, 15, 16, 19].

On top of vigorous-intensity effects, running while navigating conferred additional benefits on our measure of spatial cognition. Spatial learning and memory were tested using the Groton Maze Learning Test, which is a close 2D analog to the 3D wayfinding of orienteering. Although all groups increased in spatial learning efficiency, the vigorous orienteering group improved the most and was the only group to improve in spatial memory after a delay. It is important to consider why. One reason relates to the specific cognitive processes tested. During the Groton Maze Learning Test, participants had to recall the maze route immediately and after a 10-minute delay, requiring skills that are highly dependent on the hippocampus, a brain region that is responsive to intervention-induced plasticity [60]. A second reason why orienteering may preferentially benefit spatial cognition relates to its overlap in cognitive processes engaged by the task. In general, cognitive training effects tend to transfer more readily to “near-transfer” tasks, i.e., tasks that closely resemble the cognitive demands of the training protocol, than “far-transfer” tasks, i.e., tasks that depend on more disparate cognitive processes [61, 62]. In the case of orienteering, spatial cognition would classify as a near-transfer task and based on this framework, would be expected to benefit the most.

In contrast, the high-interference memory task would be considered a far-transfer task and, by the same logic, would be less likely to show additive effects, as was observed. Instead, high-interference memory (lure discrimination index) improved to a similar extent for both vigorous exercise and orienteering groups, suggesting that this aspect of cognition is more sensitive to the acute effects of exercise intensity than the combined effects of the exercise-cognitive training that is experienced during an acute bout of orienteering. Although the effects of vigorous exercise on high-interference memory were expected and consistent with prior work [9, 56, 57, 63], we were surprised to observe a decrement in high-interference memory performance following moderate-intensity orienteering. This may be related to the amount of exercise-induced BDNF, which is less after moderate intensities compared to vigorous [6–8]. Indeed, those who orienteered at moderate intensity produced less BDNF than those who orienteered at a vigorous intensity, and this may have reduced their neurogenic support, rendering substrate-dependent memory benefits unobtainable.

The difference in BDNF levels between moderate and vigorous intensity orienteering may also help to explain why expert taxi drivers experience a trade-off that augments their posterior (primarily relating to spatial processing) hippocampus at the cost of their anterior (mainly involved in episodic memory) hippocampus [39]. Taxi drivers are sedentary while driving, which is in stark contrast to expert orienteers who perform their sport at a rapid running speed [29]. The lack of vigorous movement during navigation may be why we see evidence for a trade-off in expert taxi drivers but not in expert orienteers. Regardless of the mechanism, we found that engaging in vigorous-intensity exercise while orienteering benefited spatial memory but not high-interference memory. Although the reasons for this are unclear, a single acute orienteering session may not be a strong enough stimulus to evoke adaptative changes in all hippocampal functions. Future research is needed to investigate whether chronic orienteering interventions can produce “far-transfer” effects beyond the effect of spatial cognition observed here.

Although BDNF increased more for the vigorous interventions than the moderate intervention, it was expected to increase even more following vigorous orienteering, but that was not observed. Our sample of healthy, recreationally active younger adults may have been the reason why. Unlike older adults, prior research with younger adults reveals no additional boost in BDNF from exercise-cognitive training, as was observed here [57, 64]. This makes sense given that BDNF is thought to respond to energetic challenges [65], and in our sample of recreationally active younger adults, the additional challenge of running while navigating may not have been enough of an acute energetic demand. This may be especially true given the wayfinding task was short (only ~12 minutes) and across a familiar terrain. Future work should examine the potentially additive effects of orienteering versus running on BDNF using longer and less familiar routes. Additionally, there is evidence that females have lower BDNF responsivity to acute exercise [66] and lower lactate responses at the same relative exercise intensity compared to males [67]. These potential sex-based differences in lactate-induced BDNF activation may be at play with our predominately female sample (65%) and should be followed up in future work.

Despite our participants’ familiarity with the campus on which the orienteering course was set, both the moderate and vigorous intensity orienteering groups travelled significantly farther and, by extension, made more errors than the most efficient route. Interestingly, the distance travelled while orienteering was associated with several of our baseline measures. Notably, those who travelled shorter distances (i.e., made fewer errors) had better spatial memory at baseline, as revealed by both self-report and task performance, which reaffirms the existence of overlapping cognitive processes engaged between navigation and spatial memory [30]. Also, those who travelled shorter distances reported greater reliance on egocentric spatial navigation. Allocentric spatial navigation was not as strongly related to course distance travelled, which was surprising given that allocentric spatial processing, like egocentric spatial processing and spatial memory, have been previously associated with expertise in the sport of orienteering [27]. The weak association between allocentric spatial processing and navigational efficiency observed here may be related to participants’ familiarity with the course terrain. We set the course on campus because it provided a safe environment for orienteering, but it is important to acknowledge that navigational tendencies may differ between familiar and unfamiliar terrains [30]. For example, participants could identify campus buildings by their names and then navigate based on previously learned routes rather than utilize allocentric spatial navigation. It will be important for future work to examine the orienteering interventions across novel terrains over a variety of course difficulties.

Moreover, overreliance on GPS devices may be a factor because it minimizes active navigation and the practice of allocentric navigation in the case of “use it or lose it” [36]. GPS may be used more commonly by those with little experience in orienteering, as allocentric navigation may require more practice to be developed [30]. Unfortunately, we did not capture GPS use, but we would recommend this be done in future studies. Furthermore, prior research suggests that females may rely less on allocentric navigation than males [68], and our sample was predominantly female. Although we did not power our sample size to examine sex differences, it is recommended that future research do so. Another reason why we failed to observe a strong association between allocentric spatial processing and navigational efficiency is speculative but worth noting; this study was conducted in North America where orienteering awareness and practice is relatively limited compared to Nordic countries where orienteering is embedded into the school curricula and local cultural activities [69]. This fact should be considered when comparing studies from different countries.

## Conclusion

This study demonstrates the effect of vigorous exercise on lactate, BDNF and hippocampal-dependent memory. It also reveals that orienteering may outperform exercise in improving spatial memory when done at a vigorous intensity. Together, this study establishes the efficacy of using orienteering to improve cognition in younger adults and provides essential groundwork for future research in older adult or AD populations to help preserve cognitive function across the lifespan.

## Supporting information

S1 Appendix Demographic questionnaire. The included questions comprised the demographics questionnaire administered in the online baseline questionnaire.
(DOCX)

S1 Data (XLSX)
