# Supplementary material for: Orienteering combines vigorous-intensity exercise with navigation to improve human cognition and increase brain-derived neurotrophic factor
Source: PLoS One. 2024 May 22;19(5):e0303785. doi: 10.1371/journal.pone.0303785 (PMC11111042; doi:10.1371/journal.pone.0303785)
Supplement: S1 Appendix — The included questions comprised the demographics questionnaire administered in the online baseline questionnaire. (DOCX) [file pone.0303785.s001.docx]

**S1 Appendix**

**Demographic Questionnaire**

1. How old are you? (required)
2. What is your biological sex at birth? (required)
3. Male
4. Female
5. Have you participated in any sports in the past? If so, please list what sports you took part in.
6. Do you currently participate in any sports? If so, please list what sports you take part in.
7. How many hours of video games do you play a week? Choose one of the following answers:
8. None
9. Less than 1 hour
10. 1 to <3 hours
11. 3 to <5 hours
12. 5 to <7 hours
13. 7 to <9 hours
14. > 9 hours
15. Prefer not to answer
16. Other (please specify how many hours) ____.
17. If you play video games, please list the types of video games you play. Otherwise, type N/A.
18. What is the highest level of education you have completed? Choose one of the following answers:
19. Less than a high school diploma
20. High school degree or equivalent
21. Bachelor’s degree (ie, BA, BS, etc.)
22. Master’s degree (ie, MA, Med, etc.)
23. Doctorate (ie, PhD, EdD, etc.)
24. Prefer not to answer.
25. Are you currently/were you a student at McMaster University?
    1. Yes
    2. No
    3. Prefer not to answer.
26. On a scale from 1-5, how familiar would you say you are with the McMaster Campus?
    1. 1 (not very familiar)
    2. 2
    3. 3
    4. 4
    5. 5 (very familiar)
27. How many times have you done orienteering in the past?
    1. None
    2. 1
    3. 2
    4. 3
    5. 4
    6. 5
    7. More than 5
